# Case Report: Concurrent thyroid tuberculosis and tuberculous lymphadenitis initially misdiagnosed as malignancy by ^18^F-FDG PET/CT

**DOI:** 10.3389/fsurg.2026.1889766

**Published:** 2026-08-12

**Authors:** Zhenyu Zhang, Huiling Wang, Yaqin Wu, Yali Lei, Chaojie Zhang

**Affiliations:** 1 Department of Breast and Thyroid Surgery, Hunan Normal University First Affiliated Hospital Hunan Provincial People's Hospital, Changsha, Hunan, China; 2 Department of Breast and Thyroid Surgery, Hunan Provincial People's Hospital (The First Affiliated Hospital of Hunan Normal University), Changsha, Hunan, China; 3 Department of Pathology, Hunan Provincial People’s Hospital (The First Affiliated Hospital of Hunan Normal University), Changsha, Hunan, China; 4 Hunan Research Institute of Geriatrics, Changsha, Hunan, China

**Keywords:** ^18^F-FDG PET/CT, case report, core needle biopsy, lymph node tuberculosis, thyroid tuberculosis

## Abstract

**Background:**

Tuberculous lymphadenitis (TBL) is a common manifestation of extrapulmonary tuberculosis (EPTB). However, establishing a definitive diagnosis can be challenging, particularly in primary healthcare settings, when the clinical presentation is atypical or when TBL coexists with another rare form of EPTB—thyroid tuberculosis (TB). Whether primary or secondary, TB remains an exceedingly uncommon clinical entity, even in the context of the persistently high overall prevalence of tuberculosis in China. Thyroid tuberculosis accounts for approximately 0.1%–0.4% of all thyroid diseases, with an even lower incidence of primary thyroid involvement. Herein, we report a case in which ^1^⁸F-FDG PET/CT imaging initially suggested malignant lesions involving thyroid nodules and cervical lymph nodes; however, the final histopathological assessment and subsequent favorable response to diagnostic anti-tubercular therapy confirmed the diagnosis of concurrent thyroid tuberculosis and tuberculous lymphadenitis.

**Case description:**

The patient was a 68-year-old male presenting with a 10-day history of a palpable neck mass. Thyroid ultrasonography revealed multiple bilateral thyroid nodules classified as TI-RADS category 4b, accompanied by markedly elevated serum anti-thyroglobulin antibody (TGAb) and anti-thyroid peroxidase antibody (TPOAb) levels. An enlarged lymph node was detected in the right cervical region. An ^1^⁸F-FDG PET/CT scan performed at an external institution demonstrated lesions with increased FDG uptake involving the thyroid gland, multiple systemic lymph nodes, and the lungs. To clarify the diagnosis, ultrasound-guided core needle biopsy (CNB) was performed on the bilateral thyroid nodules and the right cervical lymph node. Histopathological examination revealed granulomatous inflammation, and tuberculosis could not be excluded. Polymerase chain reaction (PCR) testing and fungal polysaccharide immunofluorescence (IF) staining were both negative, while acid-fast bacilli staining showed suspicious positive results. The patient exhibited no specific clinical symptoms and denied a history of exposure to tuberculosis-endemic areas. Based on these findings, diagnostic anti-tubercular therapy was initiated. A limited initial follow-up assessment demonstrated a favorable therapeutic response, supporting the diagnosis, although the patient was subsequently lost to long-term follow-up due to financial and adherence issues.

**Conclusion:**

This case highlights the importance of considering the possibility of thyroid tuberculosis in clinical practice. With the increasing detection of thyroid nodules during routine examinations, accurate differentiation between benign and malignant etiologies is crucial. Incorporating tuberculosis infection into the differential diagnosis may help prevent unnecessary surgical interventions for suspected thyroid malignancy.

## Introduction

Tuberculosis remains a major global public health concern. According to the World Health Organization Global Tuberculosis Report 2025, an estimated 10.7 million new cases of tuberculosis occur worldwide, with China ranking fourth among the 30 high-burden countries (1). Extrapulmonary tuberculosis accounts for approximately 15% to 20% of global tuberculosis cases, most frequently involving the lymph nodes, pleura, skeletal system, and genitourinary tract (2). Thyroid involvement is exceedingly rare, accounting for less than 1% even in high-prevalence regions and the estimated incidence of thyroid tuberculosis ranges from 0.1% to 0.4% of all thyroid diseases (3–5). This rarity is primarily attributed to the gland's rich vascular supply, high iodine concentration within the colloid, and the protective barrier provided by the thyroid capsule, which collectively inhibit the colonization of Mycobacterium tuberculosis (6–9).

Clinically, the incidence of thyroid tuberculosis is low, and the absence of characteristic symptoms makes its diagnosis challenging. Imaging modalities, including ultrasonography (US), computed tomography (CT), and ^1^⁸F-FDG PET/CT, often demonstrate variable findings, rendering differentiation from thyroiditis and malignant tumors difficult. A history of tuberculosis elsewhere in the body or exposure to Mycobacterium tuberculosis may aid diagnosis. Anti-tuberculosis chemotherapy can be employed for treatment; however, in the absence of pathological confirmation, misdiagnosis frequently occurs. This diagnostic uncertainty may lead to unnecessary surgical interventions that could be prevented by timely and accurate identification.

We adhered to the CARE reporting guidelines in presenting a case report describing the coexistence of thyroid tuberculosis and tuberculous lymphadenitis. ^1^⁸F-FDG PET/CT initially suggested possible malignancy of thyroid nodules and cervical lymph nodes. Following multidisciplinary team (MDT) evaluation, ultrasound-guided core needle biopsy (US-CNB), and assessment of therapeutic response to anti-tuberculosis treatment, the diagnosis of tuberculosis infection was established.

## Case presentation

### Patient information

A 68-year-old man presented with an incidental neck mass noted 10 days prior. He denied associated symptoms, including pain, fever, night sweats, hoarseness, dysphagia, or unintended weight loss. The patient had a long-term history of alcohol consumption (approximately 270 mL per day for 40 years) and smoking. In May 2025, he had been hospitalized for co-infection with Legionella pneumonia and COVID-19, from which he had fully recovered. He denied any prior history of hepatitis or tuberculosis.

### Clinical findings

Physical examination revealed visible neck asymmetry with fullness on the right side. The trachea was midline. The thyroid gland was diffusely enlarged to grade II, with multiple firm, non-tender nodules palpable bilaterally. The largest nodule measured approximately 1.5 cm × 1.2 cm. Several hard, immobile, non-tender, matted lymph nodes were palpable on the right side of the neck from the submandibular region to the supraclavicular fossa. No left-sided lymphadenopathy was detected.

## Diagnostic assessment

### Pre-admission investigations

Before admission, thyroid US performed at an external facility revealed multiple bilateral thyroid nodules with suspicious malignant features, classified as TI-RADS category 4b. The dominant nodules measured 13.3 mm in the right lobe and 7.2 mm in the left lobe. Concurrently, multiple enlarged right cervical lymph nodes were identified, the largest located at level II and measuring 47 mm × 11 mm. Ultrasound-guided fine-needle aspiration (US-FNA) of the thyroid nodules resulted in a cytological diagnosis of “Atypia of Undetermined Significance.” An ^1^⁸F-FDG PET/CT scan demonstrated hypermetabolic activity within both thyroid lobes (SUVmax 11.55), cervical (SUVmax 8.44), mediastinal (SUVmax 7.09), and retroperitoneal lymph nodes (SUVmax 8.89), as well as multiple pulmonary nodules (SUVmax 1.79), raising suspicion of a primary malignancy with extensive metastases.

### In-hospital diagnostic workup

**Figure 1:**
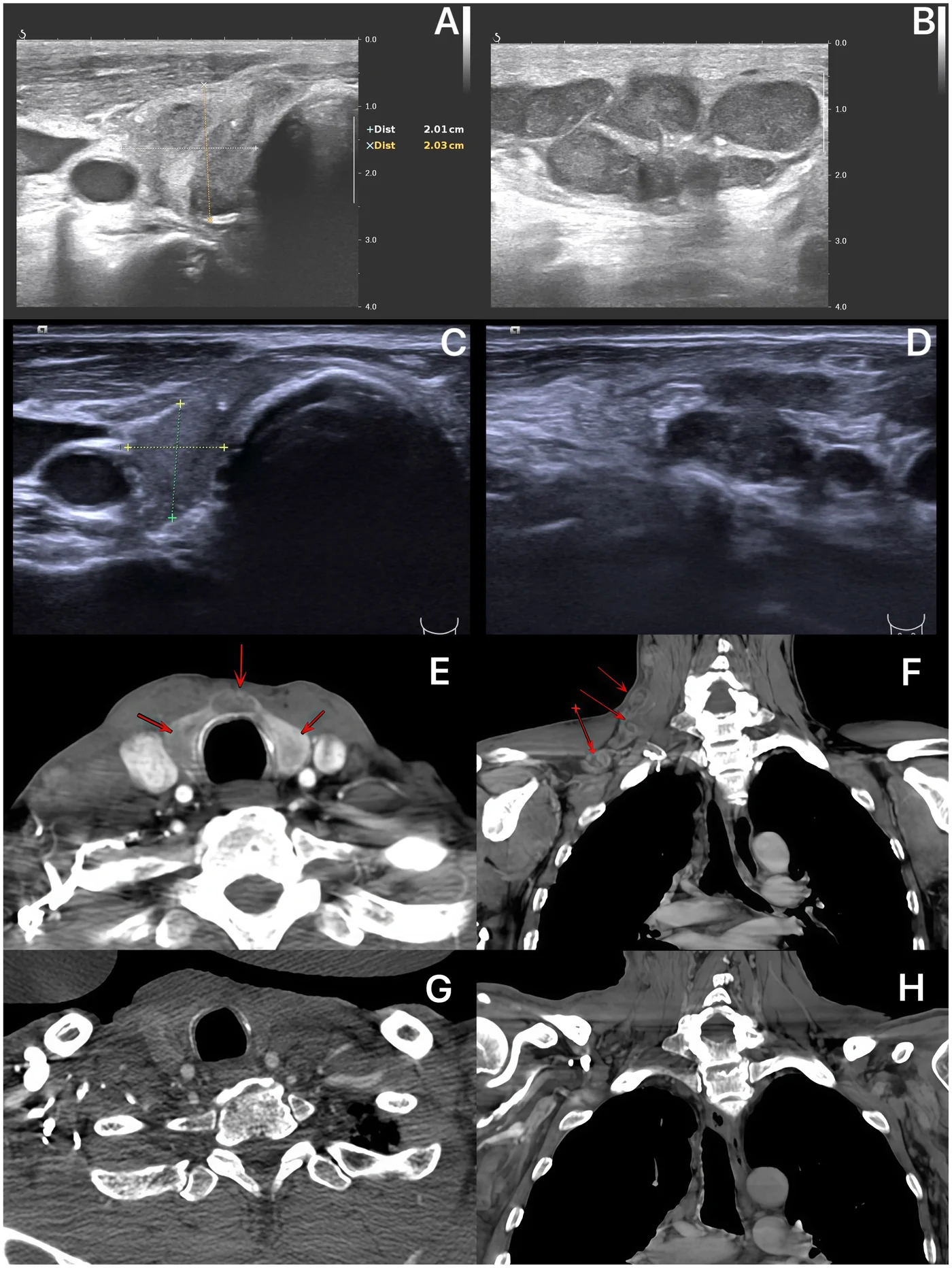
**(A,B)** Pre-treatment ultrasound of thyroid nodules and enlarged lymph nodes. **(C,D)** Post-treatment ultrasound of the thyroid and enlarged lymph nodes. **(E,F)** Pre-treatment neck/chest enhanced CT of thyroid nodules and enlarged lymph nodes. **(G,H)** Post-treatment neck/chest enhanced CT of the thyroid and enlarged lymph nodes.

In September 2025, the patient was admitted to our department for further evaluation. Laboratory testing revealed elevated anti-thyroglobulin antibody (TGAb: 35.87 IU/mL, normal range: 0–4.11 IU/mL) and anti-thyroid peroxidase antibody (TPOAb: 502.17 IU/mL, normal range: 0–5.61 IU/mL) levels. Thyroid function tests were within normal limits, as were hepatic and renal profiles. Thyroid US and neck/chest contrast-enhanced CT confirmed bilateral thyroid nodules and multiple enlarged right cervical lymph nodes (Figure 1). Findings of chronic bronchitis and pulmonary bullae were noted, but no definitive active tuberculosis lesions were identified.

**Figure 2:**
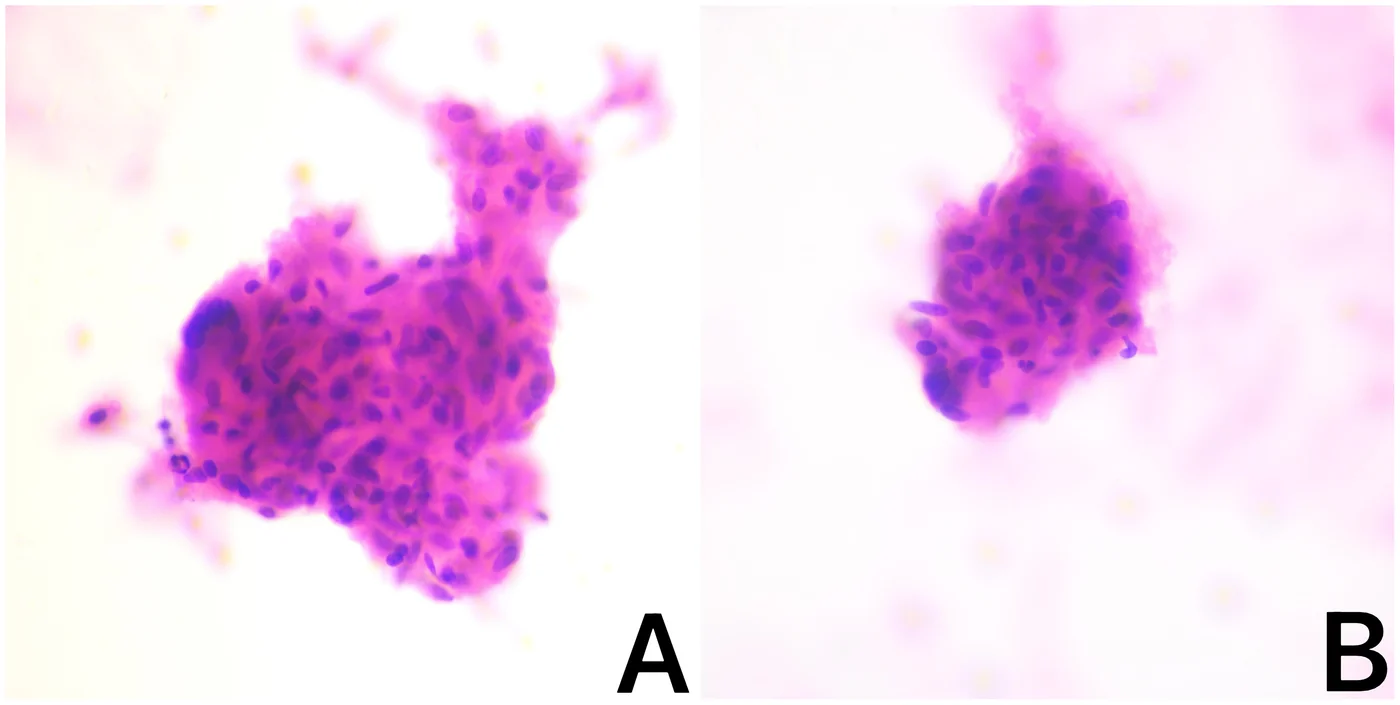
CNB image of the thyroid gland (400×, hematoxylineosin staining): epithelioid cell clusters, granulomatous inflammation could not be ruled out. **(A)** Left thyroid nodules. **(B)** Right thyroid nodules. CNB, core needle biopsy.

**Figure 3:**
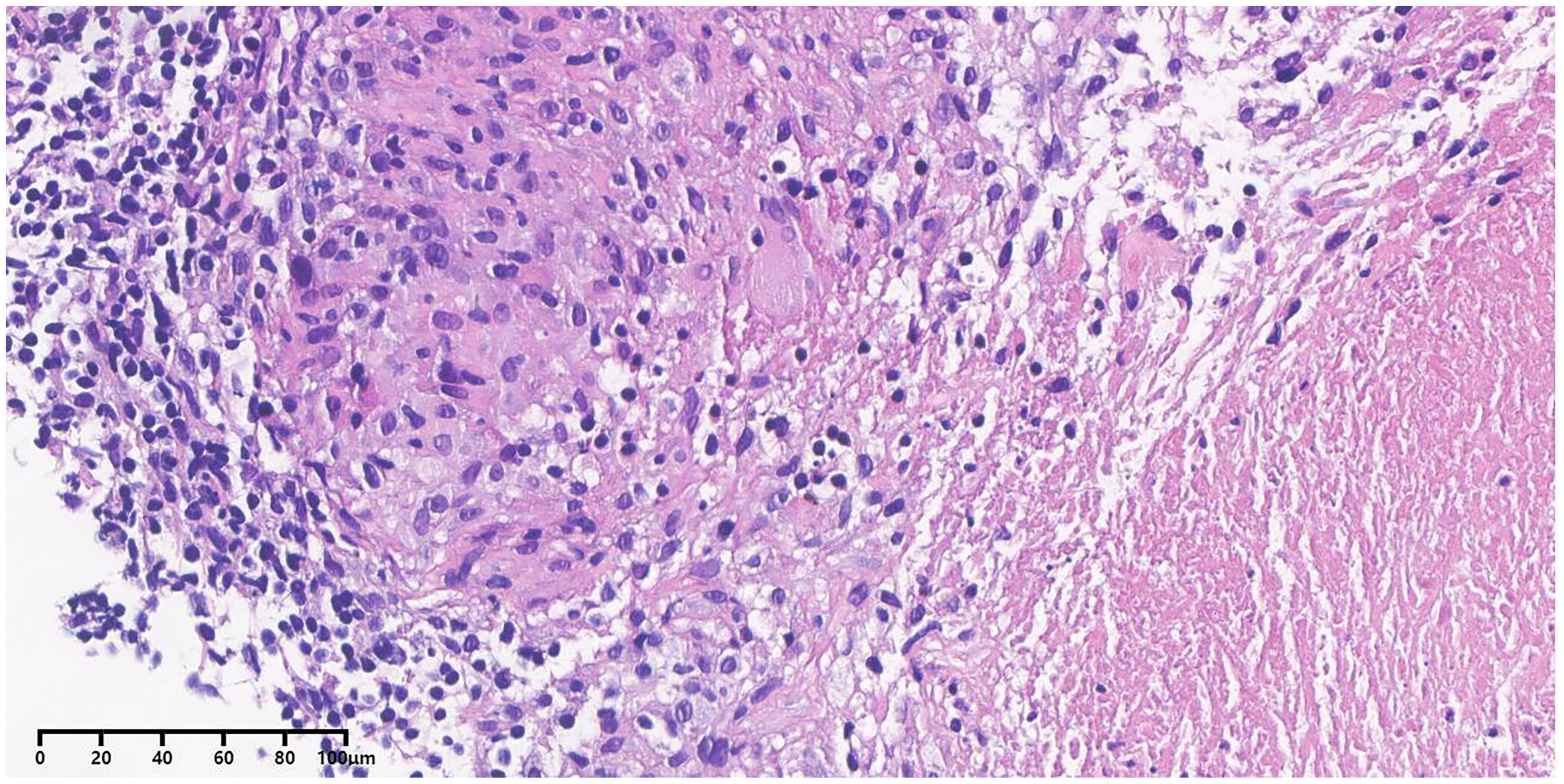
Lymph node biopsy (200×, hematoxylineosin staining): granulomatous inflammation with caseous necrosis.

As the diagnosis remained uncertain, the multidisciplinary team (MDT) reached a consensus to repeat ultrasound-guided core needle biopsy (US-CNB) targeting bilateral thyroid nodules and the right cervical lymph nodes. Histopathological examination of the thyroid nodule specimen indicated that granulomatous inflammation could not be excluded (Figure 2), while the core biopsy of the lymph nodes revealed granulomatous inflammation with caseous necrosis, highly suggestive of tuberculosis (Figure 3). Acid-fast bacillus staining showed indeterminate positivity. However, polymerase chain reaction (PCR) testing for Mycobacterium tuberculosis complex and fungal staining of the same specimen were negative. Based on the histopathological evidence of caseating granulomas, concurrent thyroid tuberculosis and tuberculous lymphadenitis were suspected.

### Therapeutic intervention

The patient was referred to a specialized tuberculosis hospital and initiated standard anti-tuberculosis therapy. The regimen consisted of a 2-month intensive phase with isoniazid (300 mg daily), rifampicin (600 mg daily), ethambutol (1,200 mg daily), and pyrazinamide (1,500 mg daily), followed by a 4-month continuation phase with isoniazid and rifampicin.

### Follow-up and outcomes

The diagnostic and therapeutic timeline is summarized in (Table 1). Follow-up assessment after treatment demonstrated normal hepatic and renal function without evidence of drug-induced toxicity. Follow-up ultrasound and contrast-enhanced CT examinations of the neck and chest were performed (Figure 1), revealing complete resolution of the thyroid nodules. Although bilateral cervical lymphadenopathy persisted, the lesion size remained stable, and some lymph nodes showed internal calcification. Imaging findings were consistent with the healing stage of tuberculous lymphadenitis and confirmed a favorable therapeutic response to empirical anti-tuberculosis therapy, establishing the final diagnosis of thyroid tuberculosis with tuberculous lymphadenitis. The patient was advised to complete the full course of anti-tuberculosis treatment and adhere to routine monitoring and follow-up, but subsequent follow-up data were unavailable. We acknowledge that the incomplete treatment and follow-up information represents a significant limitation of this case report.

**Table 1:** Diagnostic and therapeutic timeline of the patient.

Time point	Event	Key findings/outcome
Day 0	Incidental neck mass noted	No associated symptoms (pain, fever, hoarseness, weight loss)
Day 3	Thyroid ultrasound (external)	TI-RADS 4b nodules (R:13.3 mm, L:7.2 mm); enlarged right cervical LN (47 × 11 mm)
Day 5	US-FNA of thyroid nodules	Cytology: “Atypia of Undetermined Significance"
Day 7	18F-FDG PET/CT	Hypermetabolic lesions in thyroid, multiple LNs (cervical/mediastinal/retroperitoneal), and lungs—suspected metastatic malignancy
Day 14	Hospital admission	TGAb 35.87 IU/mL, TPOAb 502.17 IU/mL; thyroid function normal
Day 16	Contrast-enhanced CT	Confirmed thyroid nodules and cervical lymphadenopathy; no active TB lesions
Day 18	US-CNB (thyroid + cervical LN)	Thyroid: granulomatous inflammation (TB not excluded); LN: caseous necrosis, AFB suspicious positive; PCR negative
Day 21	MDT consensus	Initiated diagnostic anti-tuberculosis therapy
Day 28	Referral to TB hospital	Started standard anti-TB therapy (2HRZE/4HR); short-term follow-up showed reduction of nodules, but patient later lost to follow-up due to financial and adherence issues

LN, lymph node; US-FNA, ultrasound-guided fine-needle aspiration; CNB, core needle biopsy; AFB, acid-fast bacilli; PCR, polymerase chain reaction; TB, tuberculosis; MDT, multidisciplinary team.

## Discussion

Thyroid tuberculosis, a rare form of extrapulmonary tuberculosis, can be etiologically classified into primary and secondary types. The estimated incidence of thyroid tuberculosis is 0.1%–0.4% of all thyroid diseases, with a slight female predominance and peak incidence in the 20–50 year age group. Primary thyroid tuberculosis results from hematogenous dissemination of Mycobacterium tuberculosis during the initial infection, with the pathogen subsequently remaining dormant within the thyroid parenchyma. Individuals with latent infection are typically asymptomatic and do not develop active disease. Progression from latent to active tuberculosis is influenced by multiple factors, including age at infection, host immune status, and the duration since exposure. Epidemiological data indicate that approximately one-fifth of the Chinese population harbors latent tuberculosis infection, with a lifetime risk of 5%–10% for developing active disease (10, 11). Secondary thyroid tuberculosis occurs due to active tuberculous lesions at other sites spreading through the bloodstream, lymphatic system, or by direct extension.

The differential diagnosis of granulomatous inflammation of the thyroid gland encompasses a wide spectrum of etiologies, including infectious causes (tuberculosis, fungal infections), autoimmune conditions (de Quervain thyroiditis, also known as subacute granulomatous thyroiditis), granulomatous diseases (sarcoidosis), and foreign body reactions. De Quervain thyroiditis, the most common granulomatous disease of the thyroid, is a self-limiting inflammatory condition believed to be triggered by viral infection. It typically presents with a painful goiter, derangements in thyroid function tests (initially hyperthyroidism followed by hypothyroidism), and diffusely increased thyroid echogenicity on ultrasound (12). In contrast, our patient presented with painless, discrete bilateral thyroid nodules, normal thyroid function, and elevated thyroid autoantibodies—features more consistent with tuberculous involvement. Histopathologically, de Quervain thyroiditis typically shows non-necrotizing granulomas with giant cells surrounding a core of colloid (12), whereas our biopsy specimens demonstrated caseating granulomas, a hallmark of tuberculous infection. Other granulomatous diseases such as sarcoidosis may present with non-caseating granulomas and typically involve other organ systems, while fungal infections can be excluded by negative fungal staining, as in our case.

In the present case, ^1^⁸F-FDG PET/CT demonstrated intense hypermetabolic activity in the thyroid nodules (SUVmax 11.55), cervical lymph nodes (SUVmax 8.44), mediastinal lymph nodes (SUVmax 7.09), and retroperitoneal lymph nodes (SUVmax 8.89), while the pulmonary nodules showed relatively low FDG uptake (SUVmax 1.79). The markedly elevated FDG uptake in the thyroid and lymph nodes initially raised strong suspicion of disseminated malignancy, consistent with the well-recognized diagnostic pitfall that tuberculosis can mimic malignancy on PET/CT. A review of the limited literature on ^1^⁸F-FDG PET/CT in thyroid tuberculosis reveals similarly increased FDG uptake in tuberculous lesions, with SUVmax values ranging widely from 2.5 to 15.0 depending on the degree of inflammatory activity. This overlapping metabolic profile between tuberculosis and malignancy underscores the fundamental limitation of ^1^⁸F-FDG PET/CT in distinguishing infectious from neoplastic processes, particularly in extrapulmonary sites (13, 14). Therefore, while PET/CT is valuable for detecting the extent of disease, histopathological confirmation remains indispensable for definitive diagnosis in such challenging cases.

Thyroid hormones play an essential role in maintaining homeostatic immune competence during mycobacterial infection (15), and dysregulation of the thyroid axis may increase susceptibility to Mycobacterium tuberculosis. In the present case, although the patient exhibited elevated levels of TGAb and TPOAb, core needle biopsy (CNB) histology did not confirm Hashimoto's thyroiditis. Notably, emerging evidence indicates that Hashimoto's thyroiditis may influence the clinical course of tuberculosis through mechanisms involving immune dysregulation and consequent hypothyroidism. This hypothesis is supported by a nationwide longitudinal cohort study conducted by Cheng et al. (16), which demonstrated a 2.91-fold increased risk of developing tuberculosis among individuals with hypothyroidism compared with euthyroid counterparts. This association highlights the need for heightened clinical vigilance regarding infectious manifestations in patients presenting with thyroid autoantibody positivity or established hypothyroidism.

With the widespread adoption of advanced imaging modalities—including ultrasonography, computed tomography, and ^1^⁸F-FDG PET/CT—the detection rate of thyroid nodules has increased markedly, accompanied by a corresponding rise in false-positive assessments of malignancy. The diagnostic course of the present case clearly demonstrates that definitive diagnosis ultimately depends on histopathological evaluation, even in the presence of ^1^⁸F-FDG PET/CT findings that strongly suggest disseminated malignancy. In diagnostically ambiguous cases, fine-needle aspiration (FNA) is often limited by inadequate specimen quality for a conclusive diagnosis. Therefore, escalation to CNB to obtain sufficient tissue architecture, complemented by adjunctive molecular and immunological assays, is warranted when initial cytological evaluation is indeterminate.

### Strengths and limitations

The main strength of this report is the comprehensive documentation of the diagnostic journey from PET-CT suspicion of malignancy to histopathological confirmation. We acknowledge several limitations in this case. First, microbiological confirmation through mycobacterial culture was not obtained, which represents the most significant limitation. The negative PCR result may be attributed to several factors: the paucibacillary nature of extrapulmonary tuberculosis lesions, particularly in immunocompetent hosts; potential degradation of nucleic acids in formalin-fixed paraffin-embedded tissues; or the possibility that the granulomatous inflammation represented a healed or partially treated infection. Second, while the absence of mycobacterial culture confirmation prevents absolute certainty, the diagnosis of tuberculosis is strongly supported by the combination of histopathological evidence (caseating granulomas), acid-fast bacilli staining suspicious positivity, concurrent tuberculous lymphadenitis with caseous necrosis, and the favorable clinical and radiological response to anti-tuberculosis therapy. In the absence of positive cultures, a favorable response to diagnostic anti-tuberculosis therapy in conjunction with consistent histopathological findings is considered an accepted diagnostic criterion in clinical practice. Third, as a single case report, our findings require validation in larger case series.

### Take-away lessons

Thyroid tuberculosis should be considered in the differential diagnosis of thyroid nodules, especially in regions with high TB prevalence, and granulomatous inflammation of the thyroid has a broad differential diagnosis, including de Quervain thyroiditis, sarcoidosis, and fungal infections, all of which should be carefully excluded. Histopathological examination remains the diagnostic gold standard, and the combination of histopathological findings and a favorable response to anti-tuberculosis therapy can support the diagnosis even in the absence of microbiological confirmation. Although in this case, CNB provided superior diagnostic information compared with FNA for diagnosing granulomatous disease, the superiority of CNB over FNA requires further validation in larger studies.

## Patient perspective

Based on the patient's verbal description during follow-up visits, he stated: “When the PET-CT result came back suggesting cancer, I was very worried and thought I might need a major operation. I am grateful that the doctors recommended a biopsy instead of going straight to surgery. After starting the anti-tuberculosis treatment, I feel much better, and the follow-up scans showed that the nodules in my thyroid disappeared. I am relieved that I did not have to undergo a major operation.”

## Data Availability

The original contributions presented in the study are included in the article/Supplementary Material, further inquiries can be directed to the corresponding author.
